# Cytoplasmic DNA accumulation preferentially triggers cell death of myeloid leukemia cells by interacting with intracellular DNA sensing pathway

**DOI:** 10.1038/s41419-021-03587-x

**Published:** 2021-03-26

**Authors:** Tomohisa Baba, Takeshi Yoshida, Yamato Tanabe, Tatsunori Nishimura, Soji Morishita, Noriko Gotoh, Atsushi Hirao, Rikinari Hanayama, Naofumi Mukaida

**Affiliations:** 1 Division of Molecular Bioregulation, Cancer Research Institute, Kanazawa University, Kanazawa, Japan; 2 Department of Immunology, Graduate School of Medical Sciences, Kanazawa University, Kanazawa, Japan; 3 Nano Life Science Institute, Kanazawa University, Kanazawa, Japan; 4 Division of Cancer Cell Biology, Cancer Research Institute, Kanazawa University, Kanazawa, Japan; 5 Department of Transfusion Medicine and Stem Cell Regulation, Juntendo University School of Medicine, Tokyo, Japan; 6 Division of Molecular Genetics, Cancer Research Institute, Kanazawa University, Kanazawa, Japan

**Keywords:** Acute myeloid leukaemia, Chronic myeloid leukaemia

## Abstract

Accumulating evidence indicates the presence of cytoplasmic DNAs in various types of malignant cells, and its involvement in anti-cancer drug- or radiotherapy-mediated DNA damage response and replication stress. However, the pathophysiological roles of cytoplasmic DNAs in leukemias remain largely unknown. We observed that during hematopoietic stem cell transplantation (HSCT) in mouse myeloid leukemia models, double-stranded (ds)DNAs were constitutively secreted in the form of extracellular vesicles (EVs) from myeloid leukemia cells and were transferred to the donor cells to dampen their hematopoietic capabilities. Subsequent analysis of cytoplasmic DNA dynamics in leukemia cells revealed that autophagy regulated cytoplasmic dsDNA accumulation and subsequent redistribution into EVs. Moreover, accumulated cytoplasmic dsDNAs activated STING pathway, thereby reducing leukemia cell viability through reactive oxygen species (ROS) generation. Pharmaceutical inhibition of autophagosome formation induced cytoplasmic DNA accumulation, eventually triggering cytoplasmic DNA sensing pathways to exert cytotoxicity, preferentially in leukemia cells. Thus, manipulation of cytoplasmic dsDNA dynamics can be a novel and potent therapeutic strategy for myeloid leukemias.

## Introduction

Leukemia arises from the neoplastic transformation of hematopoietic stem cells (HSCs) or lineage-committed progenitor cells. The transformed cells are called leukemia initiating cells (LICs), which expand in the bone marrow and eventually spread systemically. Various types of oncogenic driver mutations have been identified in LICs even in leukemias which are morphologically classified as the same disease entity. Despite the monoclonal origin of LICs in most cases, some leukemia patients contain heteroclonal LICs simultaneously, thereby promoting leukemia progression^1^. The diversity of oncogenes generating LICs and the presence of heteroclonal LICs hinder the development of a molecular targeted therapy, which can be effective to treat various types of leukemia. Therefore, the establishment of a more effective and universal therapeutic strategy is urgently required to treat leukemia, besides molecular targeting therapy.

We have previously examined the cellular and molecular mechanisms underlying the competitive interaction between normal hematopoietic cells and leukemia cells. In the initiation step of chronic myeloid leukemia (CML) development, we demonstrated that CML LIC-derived basophil-like leukemia cells constitutively secrete an inflammatory chemokine, CCL3, which has a potent capacity to inhibit normal hematopoiesis, thereby indirectly promoting leukemia-tropic hematopoiesis in the limited bone marrow (BM) space in CML-bearing mice^2,3^. Accordingly, the inhibition of CCL3-mediated signal by the treatment with an allosteric inhibitor of CCL3 receptor, maraviroc, efficiently prevented the development of CML but failed to eradicate CML LICs^3^.

Hematopoietic stem cell transplantation (HSCT) is widely used as a curative therapy for various hematologic malignancies due to its ability to eradicate LICs. To examine the intercellular interaction between residual LICs and donor-derived normal hematopoietic cells after HSCT, we conducted BM transplantation (BMT) in CML mice. We observed that BMT mostly suppressed the recurrence of residual leukemia cells in the BM, spleen (SP), and peripheral blood (PB), probably due to the elimination of residual leukemia cells by irradiation but induced fatal pancytopenia with a long period of latency in most animals. Thus, donor cell-derived normal hematopoiesis was disturbed fatally in mice subjected to BMT after transient hematopoietic reconstitution.

Here, we further revealed that CML cells, as well as acute myeloid leukemia (AML) cells, could incorporate extranuclear DNA fragments into extracellular vesicles (EVs), which are taken up by normal hematopoietic stem/progenitor cells (HSPCs) and dampen their hematopoietic capacity. Moreover, cytoplasmic DNA can trigger DNA sensing pathway-mediated cytotoxicity in leukemia cells upon its accumulation. This accumulation arises from the inhibition of its autophagy-mediated degradation and removal, as revealed by our present observations. Thus, the manipulation of cytoplasmic dsDNA dynamics can be a novel and potent therapeutic strategy for myeloid leukemias.

## Methods and Materials

### Mice

Specific pathogen-free 6 to 8-week-old male BALB/c and C57BL/6J mice were purchased from Charles River Japan (Yokohama, Japan) and designated as WT mice. CD45.1 congenic mice of the BALB/c strain and STING^−/−^ mice of the C57BL/6J strain were obtained from Jackson Laboratories (Bar Harbor, ME, USA). All mice were kept under specific pathogen-free conditions and all the animal experiments in this study complied with the Guidelines for the Care and Use of Laboratory Animals of Kanazawa University (AP-163699). Mouse experiments were not randomized. The investigators were not blinded when separating mice into each experimental group.

### Antibodies (Abs)

The following hamster, mouse, or rat anti-mouse monoclonal Abs (mAbs) were used; anti-Alix (3A9; 634502, Biolegend, San Diego, CA, USA), anti-CD4 (RM4-5; 35-0042, TONBO Biosciences, San Diego, CA, USA), anti-CD8 (53-6.7; 35-0081, TONBO Biosciences), anti-CD9 (MZ3; 124802, Biolegend), anti-CD11b (M1/70; 35-0112, TONBO Biosciences), anti-CD45.1 (A20; 25-0453, TONBO Biosciences), anti-CD45.2 (104; 50-0454, TONBO Biosciences), anti-CD45R/B220 (RA3-6B2; 35-0452, TONBO Biosciences), anti-CD81 (Rat-2; 104901, Biolegend), anti-CD117/c-kit (ACK2; 20-1172, TONBO Biosciences), anti-GAPDH (3H12; M171-3, MEDICAL & BIOLOGICAL LABORATORIES, Nagoya, Japan), anti-γH2A.X (2F3; 613408, Biolegend), anti-IRF7 (MNGPKL; 12-5829-82, eBioscience, San Diego, CA, USA), anti-Ly-6A/E/Sca-1 (D7; 12-5981, eBioscience), anti-Ly-6G/Gr-1 (RB6-8C5; 35-5931, TONBO Biosciences), and anti-TER-119 (TER-119; 35-5921, TONBO Biosciences). Mouse lineage Ab cocktail with isotype control set, was purchased from BD Biosciences (561317, San Jose, CA, USA). Isotype-matched control IgGs for individual rat mAbs and mouse mAb were purchased from BD Biosciences. Rabbit anti-actin Ab (A5060, Sigma-Aldrich, St. Louis, MO, USA) and anti-human AIM2 (D5X7K; 12948), cGAS (D1D3G; 15102), and STING (D2P2F; 13647) were purchased from Cell Signaling (Danvers, MA, USA) and were used for western blot analysis. HRP-conjugated anti-rat IgG (405405, BioLegend), anti-rabbit IgG (7074, Cell Signaling), anti-hamster IgG (127-035-099, Jackson ImmunoResearch, West Grove, PA, USA), and anti-mouse IgG (405306, BioLegend) Abs were used as the secondary Abs for western blot analysis.

### Culture of cell lines and cytotoxic assay

Kasumi-1^4^ (JCRB1003), KG-1^5^ (JCRB0065), and BALL-1^6^ (JCRB0071) cell lines were obtained from JCRB Cell Bank (Osaka, Japan). HL-60 (human AML cell line), THP-1 (human monocytic leukemia cell line), Kasumi-1 (human AML cell line), KG-1 (human AML cell line), K562 (human CML cell line), and BALL-1 (human B lymphoblastic leukemia cell line) cells were cultured in 10% FBS RPMI-1640 medium. In the cytotoxic assay of autophagy inhibitors, cell viability was determined using a Cell Counting kit-8 (DOJINDO, Kumamoto, Japan) or by counting absolute cell numbers 48 h after treatment with each inhibitor. The cells were not tested for mycoplasma contamination.

### Establishment of knock-down (KD) cell lines

HL-60 and THP-1 cells were infected with MISSION lentivirus carrying pLKO.1 puro with shRNA constructs (STING-1: TRCN0000164628; STING2: TRCN0000135555; AIM2-1: TRCN0000107501; AIM2-2: TRCN0000107502; and cGAS: TRCN0000149984), which were purchased from Sigma-Aldrich. Each cell line infected with MISSION lentivirus carrying pLKO.1 puro and with a non-mammalian shRNA control construct was used as a shControl. Stably transfected cells were selected in culture medium supplemented with 1 μg/ml puromycin for more than 2 weeks. For the transient KD of *ATG5* gene expression, HL-60 cells were transfected with ATG5 siRNA (Santa Cruz Biotechnology, Santa Cruz, CA, USA) using GENOMONE-Si transfection reagent (Ishihara Sangyo, Osaka, Japan). As a siControl, cells were transfected with control siRNA-A (Santa Cruz Biotechnology). The KD efficiency of each gene was confirmed using quantitative real-time PCR (qRT-PCR) and western blotting analyses.

### Cell preparation from BM and SP

Total BM cells from the femoral, tibial, and humeral bones were flushed using cold magnetic activated cell separation (MACS) buffer (PBS supplemented with 2 mM EDTA and 3% FBS). Subsequently, erythrocytes were removed by density gradient centrifugation using Histopaque-1083 reagent (Sigma-Aldrich) or by hemolysis using ammonium chloride lysing buffer containing 0.826% NH_4_Cl, 0.1% KHCO3, and 0.004% EDTA-4Na. Lineage marker (CD4, CD8, CD11b, Gr-1, B220, and TER-119)^−^c-kit^+^Sca-1^+^ and lineage marker^−^c-kit^+^ cells were sorted using FACSAria Cell Sorter (BD Biosciences) and were used as LKS^+^ and LK^+^ cells, respectively. In some experiments, total BM cells were biopsied from live mice and were subjected to kinetics examination of residual leukemia cells in the BM. Total SP cells were isolated via mechanical digestion from SP, and were subsequently treated with ammonium chloride lysing buffer to remove the erythrocytes.

### Retroviral preparation

MSCV-MLL-AF9-ires-GFP vector was gifted from Akihiko Yokoyama, National Cancer Center, Tsuruoka Metabolomics Laboratory, Japan. MSCV-BCR-ABL-ires-GFP and MSCV-MLL-AF9-ires-GFP plasmids were prepared as described previously^7,8^. Retroviral packaging cells (Phoenix 293T) were transiently transfected with each plasmid using jetPRIME transfection reagent (Polyplus-transfection, New York, NY, USA) to produce retrovirus carrying MSCV-BCR-ABL-ires-GFP or MSCV-MLL-AF9-ires-GFP in the culture supernatant, which were subjected to the infection into LKS^+^ cells.

### Generation of CML and AML model

LKS^+^ cells purified from BM of WT or STING^−/−^ mice were cultured in serum-free S-Clone SF-03 medium (Sanko Junyaku, Tokyo, Japan) supplemented with 1% BSA, 100 ng/ml stem cell factor (WAKO, Osaka, Japan), 100 ng/ml thrombopoietin, 25 ng/ml *fms*-like tyrosine kinase-3 ligand (R & D systems, Minneapolis, MN, USA), 10 ng/ml IL-6 (PeproTech Cranbury, NJ, USA), and 10 ng/ml IL-3 (PeproTech) for 1 day. Cultured LKS^+^ cells were infected with the retrovirus carrying MSCV-BCR-ABL-ires-GFP or MSCV-MLL-AF9-ires-GFP using ViroMag R/L kit (OZ Bioscience, San Diego, CA, USA) to obtain LICs. The resultant LICs (BCR-ABL, 200–300 GFP^+^ cells included in 15,000 LKS^+^ cells; MLL-AF9, 50–100 GFP^+^ cells included in 30,000 LKS^+^ cells) were transplanted intravenously into sublethally irradiated recipient mice (BALB/c strain, 5.5 Gy; C57BL/6J strain, 7 Gy) along with normal BM cells (BCR-ABL, 1 × 10^6^; MLL-AF9, 3 × 10^5^) in a 200 μl volume. When AML-like splenomegaly was observed around 100 days after transplantation, BM cells were harvested from the primary AML mice and stored in liquid nitrogen. A frozen stock of primary AML cells was used for the colony formation assay. To prepare a mouse AML model, 2 × 10^5^ colony-forming cells were transplanted intravenously into sublethally irradiated secondary recipient mice, along with 1 × 10^6^ normal BM cells.

### BMT therapy

CML-bearing mice were transplanted with 2 × 10^6^ healthy WT or STING^−/−^ BM cells following x-irradiation pretreatment (BALB/c strain, 5 Gy; C57BL/6J strain, 6 Gy) 10 days after the transplantation of leukemogenic cells. To discriminate the donor cells from the recipient ones, including residual leukemia cells, CD45.1^+^ congenic mouse-derived BM cells were used as donor cells in some experiments.

### In vivo administration of autophagy inhibitors

SBI-0206965 (Sigma-Aldrich) and MRT-68921 (Sigma-Aldrich) were dissolved in DMSO and distilled water, respectively, to prepare the 10 mg/ml stock solution. AML-bearing mice were intraperitoneally injected with a dose of 20 mg/kg SBI-0206965 diluted in PBS containing 5% polyethylene glycol 400 (Sigma-Aldrich) and 5% Tween 80 (Sigma-Aldrich) or MRT-68921 diluted in PBS according to the schedule (Fig. 5a).

### Isolation of EVs from SP tissues

CML mice were irradiated with 5 Gy, 14 days after LIC transplantation. The SP tissues were harvested from normal mice, untreated leukemia mice (CML and AML), or CML mice irradiated 7 days before sacrifice. Then, they were mechanically digested in 2 mM EDTA in PBS. The cells and their debris were removed using centrifugation for 5 min at 300 × *g* and filtration using a 1.2 μm filter (Minisart 17593-K; Sartorius Stedim Biotech, Goettingen, Germany). After centrifugation for 30 min at 10,000 × *g*, EVs were subsequently isolated from the supernatant via centrifugation for 2 h at 100,000 × *g*. EVs were further purified using a 0.22 μm PVDF filter (Ultrafree; Merck Millipore, Billerica, MA, USA) after they were resuspended in HBSS buffer (Sigma-Aldrich). Isolated EVs were stored at −80 °C until use.

### Isolation of EVs from leukemia cells

LKS^+^ cells were isolated from WT or STING^−/−^ mice and were infected with a retrovirus carrying MSCV-BCR-ABL-ires-GFP or MSCV-MLL-AF9-ires-GFP. Two days after the infection, 150 GFP^+ or −^ cells were sorted using FACSAria Cell Sorter and were subsequently cultured in 1.1 ml of Methocult GF M3434. MLL-AF9^+^ colony-forming cells were harvested at day 7 and were re-plated to obtain the secondary culture, and the procedure was repeated at least two times to determine leukemic transformation. EVs were isolated from the supernatant of each 3.5 mm dish for their characterization at days 7, 10, or 12 of the colony formation assay.

### Co-culture of the primitive BM cells with EVs

LKS^+^ cells isolated from WT mice were cultured and propagated in serum-free S-Clone SF-03 medium supplemented with 1% BSA, 100 ng/ml stem cell factor, 10 ng/ml IL-6, and 10 ng/ml IL-3 for 2 days. After propagated LKS^+^ cells were subsequently co-cultured with 150 μg/ml SP EVs derived from control or irradiated CML mice for 24 h, they were subjected to the microarray analysis. Freshly isolated LKS^+^ cells and LK^+^ cells were co-cultured with EVs in serum-free S-Clone SF-03 medium supplemented with 1 % BSA and 100 ng/ml stem cell factor for 6 and 18 h, for the qRT-PCR analysis and the flow cytometric analysis of IRF7 expression, respectively.

### Extraction and measurement of double-stranded DNAs (dsDNAs) in EVs and cytoplasmic fraction

The total protein concentration of EVs isolated from SP tissues and culture supernatants was measured using a BCA Protein Assay kit (Thermo Fisher Scientific, Waltham, MA, USA). For the measurement of dsDNA concentration, total DNA was purified using NucleoSpin Tissue kit (MACHEREY-NAGEL, Duren, Germany) following the digestion of vesicular-free DNAs using 27 Kunitz U/ml DNase I (QIAGEN, Chatsworth, CA, USA) for 30 min at 37 °C and its ensuing inactivation using 10 mM EDTA for 5 min at 65 °C. Cytoplasmic DNAs were extracted using a Cell Fractionation kit (Abcam, Cambridge, MA, USA), and were purified using NucleoSpin Tissue kit. The concentration of dsDNA was measured using Qubit 4 (Thermo Fisher Scientific) and Qubit 1X dsDNA HS Assay kit (Thermo Fisher Scientific). dsDNA concentration in EVs was normalized with the determined protein concentration. In some experiments, *BCR-ABL* gene was detected using genomic PCR using a specific primer set (sense: 5′-TTC AGA AGC TTC TCC CTG ACA T-3′; antisense: 5′-TGT TGA CTG GCG TGA TGT AGT TGC TTG G-3′)^9^.

### RNA isolation, cDNA synthesis, and qRT-PCR

Total RNA was isolated from cells using an RNeasy Mini Kit (QIAGEN) and then reverse-transcribed using the SuperScript IV VILO (Thermo Fisher Scientific). qRT-PCR was performed using StepOne Real-time PCR system (Thermo Fisher Scientific), using the Fast SYBR Green Master Mix (Thermo Fisher Scientific), and specific primer sets for mouse *Gapdh* gene (sense: 5′-GCG GCA CGT CAG ATC CA-3′; antisense: 5′-CAT GGC CTT CCG TGT TTC CTA-3′), mouse *Isg20* gene (sense: 5′-ACA TCC AGA ACA ACT GGC GG-3′; antisense: 5′-TGA GGA GTG GCA GCT TCT AAC-3′), mouse *Rsad2* gene (sense: 5′-TGC CTG AAT CTA ACC AGA AGA TGA A-3′; antisense: 5′-TTC TTC CAC GCC AAC ATC CA-3′), mouse *Ifit3* gene (sense: 5′-CCA TCA TGA GTG AGG TCA ACC G-3′; antisense: 5′-CAT TGT TGC CTT CTC CTC AGA GT-3′), mouse *Irf7* gene (sense: 5′-GTG TAC GAA CTT AGC CGG GA-3′; antisense: 5′-GGT TTG GAG CCC AGC ATT TT-3′), mouse *Cxcl10* gene (sense: 5′-CCA CGT GTT GAG ATC ATT GCC-3′; antisense: 5′-GAG GCT CTC TGC TGT CCA TC-3′), human *AIM2* gene (sense: 5′- AAG AAG GCA AGC AGG AGA TG-3′; antisense: 5′-TCA GCG GGA CAT TAA CCT TT-3′), human *cGAS* gene (sense: 5′-GGC GGT TTT GGA GAA GTT G-3′; antisense: 5′-TCA TAG TAG CTC CCG GTG TTC-3′), human *GAPDH* gene (sense: 5′-GCC AAA AGG GTC ATC TC-3′; antisense: 5′-TGA GTC CTT CCA CGA TAC CA-3′), human *IL-12* gene (sense: 5′-GGT ATC ACC TGG ACC TTG GA-3′; antisense: 5′-GCT TAG AAC CTC GCC TCC TT-3′), human *ISG54* gene (sense: 5′-TGC GTG AAG AAG GTG AAG AG-3′; antisense: 5′-GCA GGT AGG CAT TGT TTG GT-3′), human *ISG56* gene (sense: 5′-GCC CAG ACT TAC CTG GAC AA-3′; antisense: 5′-TTT CCT CCA CAC TTC AGC AA-3′), human *MCP-1* gene (sense: 5′-ATA GCA GCC ACC TTC ATT CC-3′; antisense: 5′-GCT TCT TTG GGA CAC TTG CT-3′), human *STING* gene (sense: 5′-CAT CGG ATA TCT GCG GCT GA-3′; antisense: 5′-TCC AGG AAG CGA ATG TTG GG-3′), and human *TNF-α* gene (sense: 5′-GGC GTG GAG CTG AGA GAT AA-3′; antisense: 5′-GAT GGC AGA GAG GAG GTT GA-3′). The relative expression of each gene was analyzed using the ΔΔCt method relative to the Ct value of the *Gapdh* gene.

### Flow cytometry

Isolated hematopoietic cells and leukemia cells were stained with various combinations of fluorescent dye-conjugated Abs. Intracellular IRF7 and intranuclear γH2A.X were stained with PE-conjugated anti-IRF7 mAb and APC-conjugated anti-γH2A.X mAb using the Foxp3/Transcription Factor Buffer Set (eBioscience). For the detection of apoptosis, cell cycle status, and total reactive oxygen species (ROS) in AML cells, cells were stained using Annexin V-FITC Apop kit (Thermo Fisher Scientific), Vybrant DyeCycle Green (Thermo Fisher Scientific), and Total ROS/Superoxide Detection kit (Enzo Life Sciences, Farmingdale, NY, USA) respectively. The expression of each molecule was determined using a FACSCantoII (BD Biosciences) and analyzed using FlowJo software (Tree Star, Ashland, OR, USA).

### Characterization of EVs

Spleen tissues were lysed in RIPA buffer supplemented with a protease inhibitor cocktail (WAKO) on ice. After centrifugation at 14,000 × *g* for 30 min, the supernatants were used as a SP tissue lysate. The protein concentration of EVs and SP lysate were determined using a BCA Protein Assay kit (Thermo Fisher Scientific). A total of 2 μg of EVs and SP lysate were lysed in 20 µL of 2× SDS sample buffer (4% SDS, 20% glycerol, 0.02% bromophenol blue, 100 mM Tris-HCl (pH 6.8)) at room temperature for 30 min, and were electrophoresed in a 5–20% polyacrylamide gel at 200 V. The protein on the gel was transferred onto a polyvinylidene fluoride membrane (Bio-Rad Laboratories, Hercules, CA, USA). The membrane was incubated in PBS supplemented with 5% skim milk and 0.05% Tween 20 for 1 h, each primary Ab for 2 h, and each HRP-conjugated secondary Ab for 1 h. The protein expression was detected using SuperSignal West Pico Chemiluminescent Substrate (Thermo Fisher Scientific) and ImageQuant LAS4000min (GE Healthcare, Chicago, IL, USA). For nano tracking analysis (NTA), EVs were diluted at 300 ng/mL with PBS, and their size distribution and particle concentration were measured using the NanoSight™ LM10 nanoparticle tracking system (Malvern Panalytical, Malvern, UK).

### Colony formation assay

Two hundred LKS^+^ cells were isolated from WT or STING^−/−^ mice and were cultured with or without 50 μg/ml SP-derived EVs in 1.1 ml of Methocult GF M3434 (STEMCELL technologies, Vancouver, BC, Canada). The number of colonies and total cells was determined 7 and 10 days after incubation, respectively. In some experiments, 200 LKS^+^ cells or 1,250 mouse AML cells were cultured with or without 5 μM SBI-0206965 in 1.1 ml of Methocult GF M3534 (STEMCELL technologies). The number of colonies and total cells was determined 7 days after incubation. A wide-area continuous image of colony formation in a 35 mm dish was obtained using BZ-X700 microscope (Keyence, Osaka, Japan) and was analyzed using Keyence Analysis Software.

### Blood test

Whole blood samples were collected using K2 EDTA-coated micro-hematocrit capillaries (VITREX, Herlev, Denmark). The number of WBCs, red blood cells (RBCs), platelets (PLTs), hemoglobin (HGB) concentration, and hematocrit (HCT) values were determined using an automatic hematology analyzer (Celltacα MEK-6358; NIHON KOHDEN CORPORATION, Tokyo, Japan).

### Microarray analysis

Total RNA was extracted and its quality was confirmed using an Agilent 2200 TapeStation (Agilent Technologies, Santa Clara, CA, USA). RNA Integrity Number (RIN) of all samples was 10.0, and the samples were subjected to microarray analysis according to the manufacturer’s instructions. In brief, 100 ng RNA samples were labeled with cyanine 3-CTP using the Low Input Quick Amp Labeling Kit, one color (Agilent Technologies). Hybridization was performed using the Gene Expression Hybridization Kit (Agilent Technologies). cRNA samples (600 ng) were subjected to fragmentation (30 min at 60 °C), and then hybridized on SurePrint G3 Mouse Gene Expression v2 8x60K Microarray Kit (G4852B, design ID: 074809, Agilent Technologies) in a rotary oven (10 rpm at 65 °C for 17 h). Slides were washed in Agilent Gene Expression Wash Buffers 1 and 2 (Agilent Technologies) and scanned using an Agilent DNA Microarray Scanner (G2600D, Agilent Technologies). The obtained data were normalized by adjusting the differences in the probe intensity distribution across different arrays as follows: the control probes were removed and the scaling factor for each array was calculated by multiplying the expression values by the scaling factor of the array where the probes were. The scaling factor was 2500 divided by the average value of probes in addition to the upper and lower 2% of the array. When there were multiple probes for a gene, the probe for which the value of the CML sample was the highest was exploited for subsequent analysis. Gene set enrichment analysis (GSEA) was performed using GenePattern server^10–12^. As the metric for ranking genes, log2_ratio_of_means was exploited, and the gene set size was set to 20 to 200. The database gene sets used in this study were hallmark and c2.cgp.v6.0.symbols.gmt.

### Statistical analysis

We did not use specific sample size calculation methods. Any technically validated data were not excluded. Data were analyzed statistically using GraphPad Prism software (Ver. 6; La Jolla, CA, USA) using the methods indicated in the legend of each figure. Two-sided Student’s *t*-test and one-way ANOVA followed by Tukey-Kramer post-hoc test were used to compare the data among two and more than two groups, respectively. Log-rank test was used to evaluate the survival curve data. A *p* < 0.05 was considered statistically significant.

## Results

### Leukemia SP-derived EVs inhibit the hematopoietic capacity of normal HSPCs

**Fig. 1:**
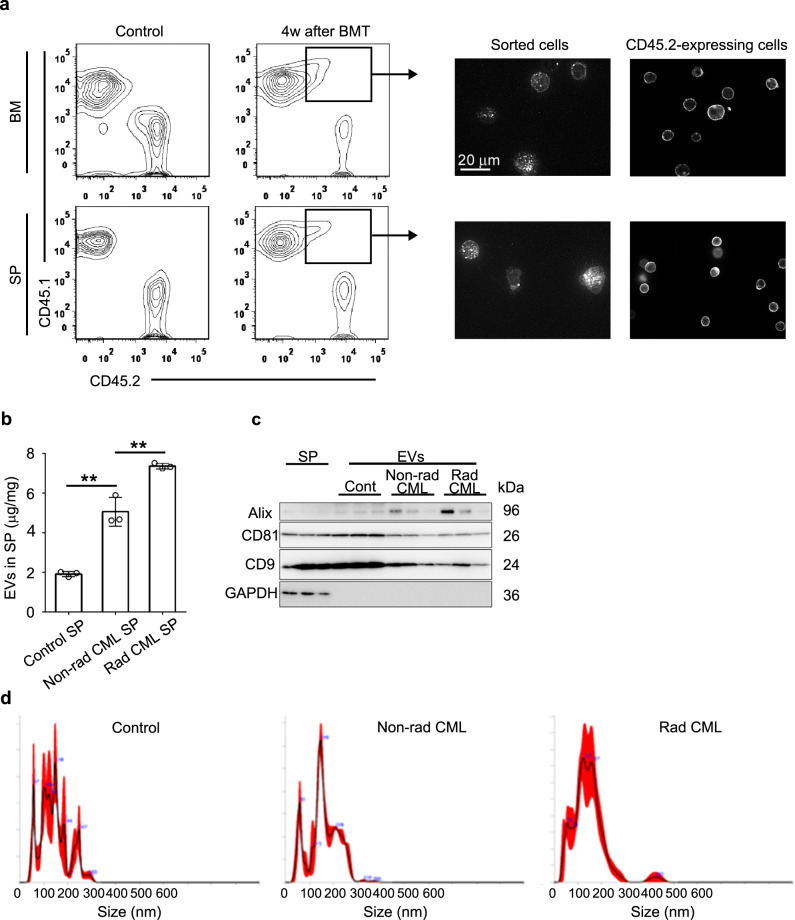
Characterization of CML SP-derived EVs. (**a**) CD45.1 and CD45.2 expression on the BM cells and splenocytes in CML-bearing mice was determined 4 weeks after BMT therapy and is shown in the left panels. As a control, cells derived from CD45.1^+^ and CD45.2^+^ mice were mixed immediately before staining with molecule-specific Abs. Representative immunofluorescence images for CD45.2 on the sorted CD45.1^+^CD45.2^+^ cells (inside square gates) from three independent experiments are shown in the right panels. Expression pattern of CD45.2 on the cells derived from CD45.2^+^ mice are shown as a control. (**b**) Protein concentration of SP-derived EVs. Data represent the mean ± SD from three independent experiments. **P < 0.01 using Tukey-Kramer test. (**c**) EV surface marker proteins (CD9 and CD81), an EV cytosolic marker protein (Alix), and a non-EV protein (GAPDH) were detected using western blot. Each lane represents an individual sample (*n* = 3). (**d**) Size distribution of control EVs, non-rad CML EVs, and rad CML EVs were analyzed using NTA. Representative results from three independent experiments are shown here.

**Fig. 2:**
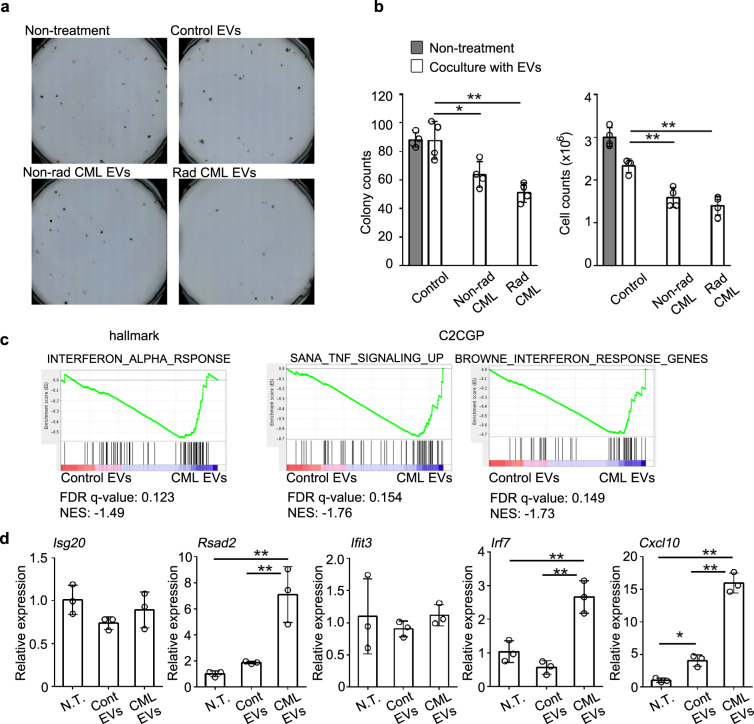
CML mouse-derived EVs disturb normal hematopoiesis. **a** Colony formation of LKS^+^ cells when they were co-cultured with or without EVs. Representative results from four independent experiments are shown here**. b** The number of colonies and total cells are shown in the left and right panels, respectively. Data represent mean ± SD from four independent experiments. **c** Gene expression was determined in LKS^+^ cells co-cultured with EVs and differentially regulated gene sets (false discovery rate (FDR) < 0.25) were identified with GSEA using hallmark and C2CGP gene sets. FDR and normalized enrichment score (NES) values were calculated from a single-sample experiment. **d** Relative mRNA expression of the indicated genes in LKS^+^ cells co-cultured with or without (N.T.) EVs. Data represent mean ± SD from three independent experiments. **P < 0.01; *P < 0.05 using Tukey-Kramer test.

CML-bearing mice underwent BMT after irradiation (5 Gy) (Supplementary Fig. 1a), and donor cell-derived hematopoietic reconstitution, but not leukemia relapse, was observed by 8 weeks after BMT (Supplementary Fig. 1b). However, most of them eventually died later than 10 weeks after BMT (Supplementary Fig. 1c) without leukemia relapse, as evidenced by few BCR-ABL^+^ cells in the BM, SP, and PB (Supplementary Fig. 1d). However, marked reductions in RBCs, HGB concentration, HCT, and PLTs were observed in the PB (Supplementary Fig. 1e). These observations suggest that donor cell-derived hematopoiesis was fatally disturbed in CML mice treated with BMT. Moreover, a substantial proportion of CD45.1^+^ donor cells in BM and SP expressed a recipient marker, CD45.2, 4 weeks after BMT (Fig. 1a), together with the punctate fluorescent signals on their cell surface (Fig. 1a), suggesting the adhesion of EVs. Indeed, CML SP contained a larger amount of EVs than control SP, and their contents were further increased by irradiation (Fig. 1b). Furthermore, all isolated EVs exhibited EV marker proteins Alix, CD81, and CD9 (Fig. 1c), and their sizes ranged from 50 to 300 nm (Fig. 1d). Either irradiated or non-irradiated CML-derived EVs reduced the colony and whole cell numbers in the colony formation assays using normal LKS^+^ HSPCs, compared with control EVs (Fig. 2a, b). Likewise, AML SP-derived EVs inhibited the colony formation of normal HSPCs (Supplementary Fig. 2), similar to CML-derived EVs. Thus, leukemia EVs reduced the hematopoietic capabilities of normal HSPCs. Additional comprehensive gene expression analysis and subsequent GSEA identified only three differentially regulated gene sets in HSPCs treated with CML EVs, and two of them were type I IFN-responsive gene sets (Fig. 2c). qRT-PCR validated the enhanced expression of *Rsad2*, *Irf7*, and *Cxcl10*, among the top five upregulated genes, which are common to two type I IFN-related gene sets in HSPCs (Fig. 2d), suggesting type I IFN signaling pathway activation by CML EVs.

### Horizontal transfer of leukemia cell-derived DNA via EVs disturbed normal hematopoiesis by activating cytosolic DNA-sensor signaling

**Fig. 3:**
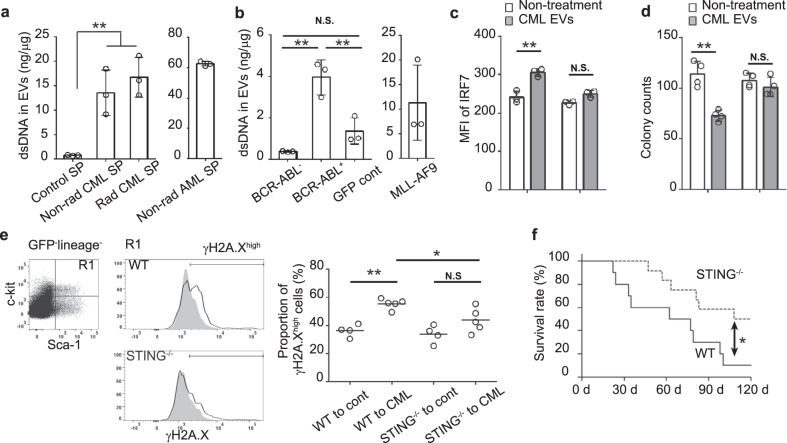
STING pathway sensing leukemia cell-derived dsDNA damages normal HSPCs. **a**, **b** dsDNA concentration in the SP-derived EVs (**a**) and in the 12-day-cultured cell-derived EVs (**b**). Data represent mean ± SD from three independent experiments. **c** Protein expression of IRF7 in LK^+^ cells when they were co-cultured with or without EVs. Mean fluorescence intensity (MFI) of cells stained with PE-conjugated anti-IRF7 mAb is shown. Data represent mean ± SD from three independent experiments. **d** Colony formation of LKS^+^ cells when they were co-cultured with or without EVs. Data represent mean ± SD from four independent experiments. **e** Expression of γH2A.X in GFP^−^LKS^+^ cells (R1 region in the right panel) 2 weeks after BMT in CML-bearing mice. As a control, normal mice were subjected to BMT. Representative results from 4 to 5 independent animals are shown in the middle panels. Gray-filled and open histograms in the left panels show control and CML-bearing mice, respectively. Each symbol represents an individual mouse (control, *n* = 4; CML, *n* = 5). **f** Survival rates within 120 days after BMT (WT, *n* = 10; STING^−/−^, *n* = 12). **P < 0.01; *P < 0.05; N.S., no significant difference using Tukey-Kramer test or log-rank test.

As the cytoplasmic double-stranded (ds)DNA fragments can robustly activate type I IFN signaling by acting on several DNA sensors^13^, we determined dsDNA content in EVs. Indeed, irradiated and non-irradiated CML SP-derived EVs contained more dsDNA fragments than control SP-derived ones (Fig. 3a) in the presence of *BCR-ABL* gene (Supplementary Fig. 3). Moreover, BCR-ABL-transformed cell-derived EVs contained a larger amount of dsDNA fragments than GFP-transduced cell-derived EVs (Fig. 3b). Likewise, the EVs present in the SP or in the culture supernatants of MLL-AF9-transformed cells contained abundant dsDNAs (Fig. 3a, b). Moreover, dsDNA was detected in EVs derived from AML mouse serum, but not in those derived from control-treated mouse serum (Supplementary Fig. 4). As multiple DNA sensors can recognize cytoplasmic DNAs to converge on a single adaptor molecule, STING, thereby activating type I IFN signaling^13^, we next examined the roles of STING pathways in this process using STING^−/−^ cells. The treatment with CML EVs significantly enhanced IRF7 protein expression in WT HSPCs, but not in STING^−/−^ cells (Fig. 3c). Moreover, the suppressive effect of CML-derived EVs on colony formation of normal HSPCs was mostly abrogated by STING deficiency (Fig. 3d). Upon BMT of CML-bearing mice, WT but not STING^−/−^ donor cells displayed an enhanced expression of a marker for DNA damage, γH2A.X, in the HSPCs, after 2 weeks (Fig. 3e). Furthermore, fatal anemia development was significantly attenuated when STING^−/−^ cells were used as donor cells (Fig. 3f). Thus, dsDNA fragments contained in leukemia cell-derived EVs damaged normal HSPCs in a cytosolic DNA-sensor signaling pathway-dependent manner both in vivo and in vitro.

### Pharmaceutical inhibition of autophagosome formation induces an accumulation of cytoplasmic dsDNA and cytotoxicity in leukemia cells

**Fig. 4:**
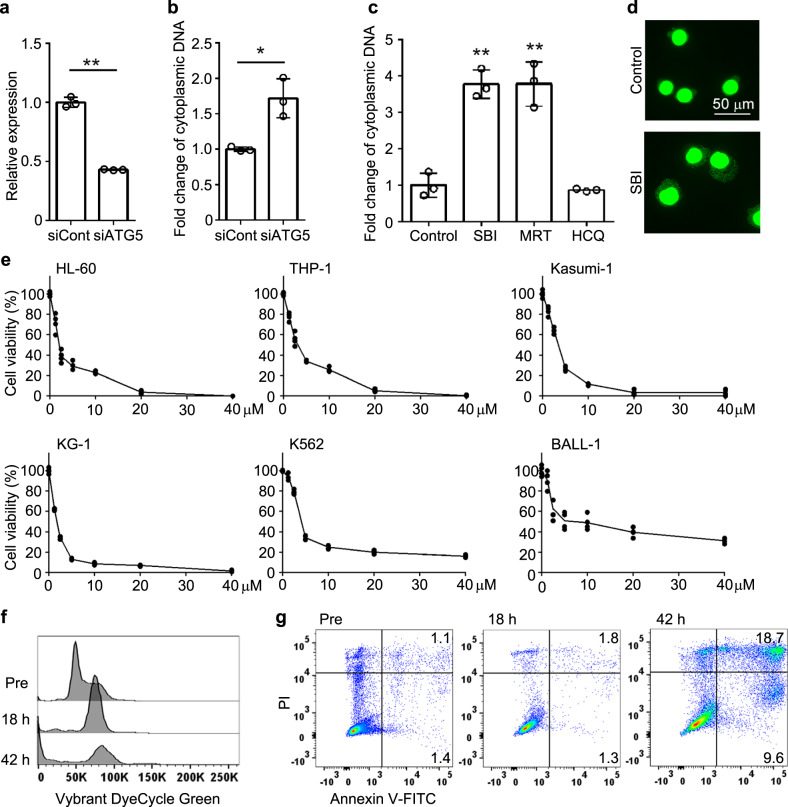
Inhibitors of autophagosome formation induce the accumulation of cytoplasmic dsDNA and cytotoxicity in leukemia cells. **a** Relative mRNA expression of *ATG5* in HL-60 cells 48 h after transfection of control or ATG5 siRNA. Data represent mean ± SD from three independent experiments. **b** Cytoplasmic dsDNA in HL-60 cells at 48 h after transfection of control or ATG5 siRNA. Fold change was calculated by dividing with the mean value of control. Data represent mean ± SD from three independent experiments. **P < 0.01; *P < 0.05 using two-sided Student’s *t*-test. **c** Cytoplasmic dsDNA in HL-60 cells 18 h after treatment with 20 μM SBI-0206965 (SBI), 2.5 μM MRT-68921 (MRT), and 20 μM hydroxychloroquine (HCQ, Sigma-Aldrich). Cells treated with DMSO were used as a control. Fold change was calculated by dividing with mean value of control. Data represent mean ± SD from three independent experiments. **P < 0.01 using Tukey-Kramer test. **d** Cytoplamic DNA was stained with Vybrant DyeCycle Green 18 h after treatment with SBI-0206965. **e** Cytotoxic assay of SBI-0206965 against leukemia cell lines (*n* = 4). **f**, **g** Cell cycle status (**f**) and apoptosis induction (**g**) was determined on HL-60 cells 18 and 42 h after treatment with SBI-0206965. Percentages of Annexin V^+^PI^−^ and Annexin V^+^PI^+^ cells in SP are shown here. Representative results from three independent experiments are shown here.

We next examined the extranuclear DNA dynamics in leukemia cells. BCR-ABL^+^ cells secreted EVs containing dsDNA at 10 days but not at 7 days of culture, when cytoplasmic DNA speckle formation was discerned (Supplementary Fig. 5a, b). Additionally, dsDNA concentration in EVs was reduced by rapamycin, which can induce the digestion of the cytoplasmic DNA by autophagic induction^14^, while the intravesicular dsDNA contents were reciprocally increased by SBI-0206965, an autophagosome formation inhibitor (Supplementary Fig. 5c). These observations indicate that autophagy can regulate cytoplasmic dsDNA accumulation and the subsequent redistribution into EVs. It was previously reported that cytoplasmic DNA activates DNA-sensor signaling in autophagy-deficient cells^14^. As leukemia EV-derived cytoplasmic DNA damages normal HSPCs (Fig. 3), we assessed whether autophagy blockade can induce cytotoxicity in leukemia cells by increasing the levels of cytoplasmic DNA. Indeed, cytoplasmic dsDNA was significantly increased in HL-60 cells, which were defective in autophagy arising from the transfection with siRNA against *ATG5* gene, a gene essentially involved in autophagy (Fig. 4a, b), in line with the previous study^14^. Moreover, HL-60 cells exhibited cytoplasmic DNA accumulation when they were treated with SBI-0206965 and MRT-68921, autophagosome formation inhibitors, whereas an autolysosome formation inhibitor, hydroxychloroquine, did not induce cytoplasmic DNA accumulation, together with an increased autophagosome formation (Fig. 4c, d, and Supplementary Fig. 6). Furthermore, SBI-0206965 or MRT-68921 efficiently induced cell death in various types of human leukemia cell lines (Fig. 4e and Supplementary Fig. 7a) with sequential induction of cell cycle arrest (Fig. 4f) and apoptotic cell death (Fig. 4g), probably due to the cytotoxic activity of cytoplasmic dsDNA. On the contrary, cytotoxicity was not marked when leukemia cells were treated with hydroxychloroquine (Supplementary Fig. 7b).

### Autophagosome formation inhibitor induces STING pathway-mediated cytotoxicity in leukemia cells

**Fig. 5:**
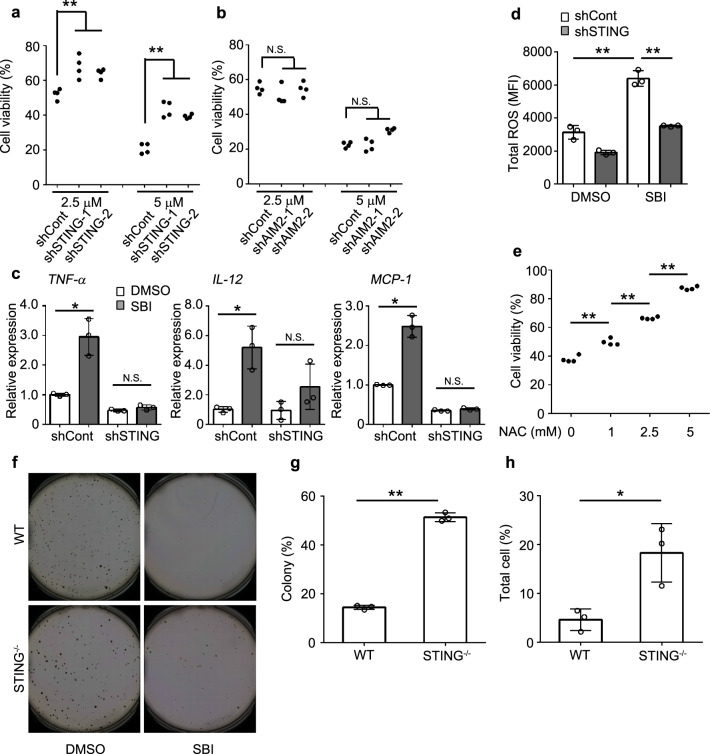
STING pathway contributes to the cytotoxicity arising from the inhibition of autophagosome formation. **a** Determination of cytotoxicity of SBI-0206965 against shRNA control or STING KD HL-60 cells (*n* = 4). **b** Determination of cytotoxicity of SBI-0206965 against shRNA control or AIM2 KD HL-60 cells (*n* = 4). **c** Relative mRNA expression of the indicated genes in control or STING shRNA-transfected HL-60 cells at 18 h after treatment with 5 μM SBI-0206965. As a control, cells were treated with DMSO. Data represent mean ± SD from three independent experiments. **d** Total ROS induction in control or STING shRNA-transfected HL-60 cells at 20 h after treatment with 5 μM SBI-0206965. Cells treated with DMSO were used as a control. MFI of cells stained using a total ROS/superoxide detection kit is shown. Data represent mean ± SD from three independent experiments. **e** Cytotoxic assay when HL-60 cells were co-cultured with 5 μM SBI-0206965 in the presence or absence of N-acetyl-L-cysteine (NAC, Sigma-Aldrich) (*n* = 4). **f** Colony formation of WT mice or STING^−/−^ mice AML cells when they were co-cultured with 5 μM SBI-0206965. Cells treated with DMSO were used as a control. **g**, **h** Suppression of colony formation of mouse AML cells by SBI-0206965 was calculated in terms of colony (**g**) and total cell number (**h**) by normalization with DMSO control for each. Data represent mean ± SD from three independent experiments. **P < 0.01; *P < 0.05; N.S., no significant difference using Tukey–Kramer test or two-sided Student’s *t*-test.

As STING pathway mainly contributed to cytoplasmic DNA-mediated damage of normal HSPCs, we examined its involvement in autophagy inhibitor-mediated cytotoxicity in leukemia cells, using STING KD HL-60 cell lines (Supplementary Fig. 8a). Both SBI-0206965 and MRT-68921 efficiently reduced cell viability in control shRNA transduced HL-60 cells, but the reduction was significantly reversed in STING KD cell lines (Fig. 5a and Supplementary Fig. 9a). STING shRNA treatment also reduced SBI-0206965-mediated cytotoxicity in another leukemia cell line, THP-1 (Supplementary Fig. 9b). Moreover, SBI-0206965-mediated cytotoxicity was significantly reduced by shRNA treatment against cyclic GMP-AMP synthase (cGAS), a major DNA sensor in STING pathway (Supplementary Fig. 10), but not against another cytosolic DNA sensor, AIM2 (Fig. 5b and Supplementary Fig. 8b). Furthermore, among the genes known to be expressed in the STING pathway, the expression of *TNF-α*, *IL-12*, and *MCP-1* genes was enhanced by SBI-0206965 in the control but not in the STING KD cells (Fig. 5c), further supporting the assumption that the STING pathway was activated by cytoplasmic dsDNA accumulation induced by autophagosome formation inhibition. Moreover, consistent with the previous report that STING pathway activation can induce ROS production, and eventually induce cellular senescence by activating the DNA damage response^15^, SBI-0206965 treatment increased intracellular ROS levels in the control but not in STING KD cells (Fig. 5d). Additionally, N-acetyl-L-cysteine, an ROS inhibitor, efficiently attenuated SBI-0206965-induced cytotoxicity in a dose-dependent manner (Fig. 5e), suggesting the involvement of STING pathway-induced ROS in autophagosome formation inhibitor-mediated cytotoxicity in leukemia cells. SBI-0206965 reduced the colony formation ability of WT mouse-derived AML cells to a larger extent than normal HSPCs (Fig. 5f and Supplementary Fig. 11), whereas the reduction was attenuated in STING^−/−^ mouse-derived AML cells (Fig. 5f, g, h).

### Autophagosome formation inhibitors can suppress the progression of AML

**Fig. 6:**
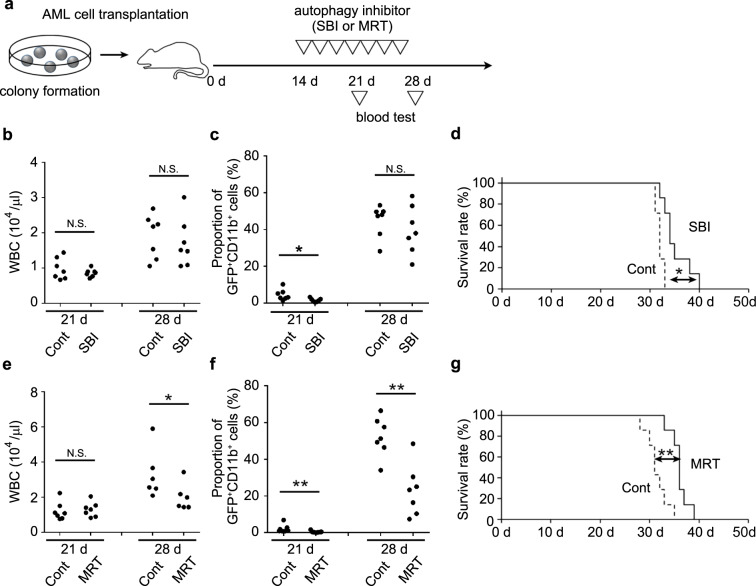
In vivo administration of autophagy inhibitors retarded the progression of AML. **a** Schematic representation of the experimental procedure of autophagy inhibitor treatment. As a control, mice were treated with each vehicle. **b**, **e** Total WBC count in mice treated with SBI-0206965 (**b**) or MRT-68921 (**e**) at the indicated time points. Each symbol represents an individual mouse (*n* = 6 or 7). **c**, **f** Percentages of GFP^+^CD11b^+^ AML cells in the PB of mice treated with SBI-0206965 **(c)** or MRT-68921 **(f)** at the indicated time points. Each symbol represents an individual mouse (*n* = 7). **d**, **g** Alteration of the survival rates of mice treated with SBI-0206965 (**d**) or MRT-68921 (**g**) (*n* = 7). **P < 0.01; *P < 0.05; N.S., no significant difference using Mann-Whitney u test or log-rank test.

Inspired by the observation that mouse normal HSPCs were more resistant than mouse AML cells to SBI-0206965, we examined its therapeutic effect. AML-bearing mice were administered the agent every other day starting from 14 days after AML cell transplantation (Fig. 6a) when leukemia cells could be apparently discerned in PB. SBI-0206965 was only partially effective to prevent the increases in total WBCs and leukemia cells in the PB (Fig. 6b, c), but significantly improved the survival rate (Fig. 6d). Moreover, when AML-bearing mice were treated with MRT-68921, with a lower IC50 against its molecular target, ULK-1, than SBI-0206965, both total WBCs and leukemia cells were efficiently reduced in the PB (Fig. 6e, f) with an improved survival rate (Fig. 6g). Thus, autophagosome inhibitors can be potent therapeutic agents against AML.

## Discussion

We revealed that the DNA fragments contained in EVs are horizontally transferred to normal hematopoietic cells to reduce their hematopoietic capabilities through the activation of cytosolic DNA-sensor pathway after BMT therapy for CML. We assumed that irradiation at BMT could enhance DNA fragmentation and subsequent EV formation. In contrast with our hypothesis, non-irradiated CML SP contained EVs, which inhibited hematopoietic colony formation to a similar extent as irradiated ones did. Hence, CML cell-derived EVs may be able to promote CML development by counteracting normal hematopoiesis. These effects may be concealed by the rapid division of primary leukemia cells but may emerge when leukemia cell growth is severely suppressed by irradiation. We further uncovered that AML cells abundantly secreted EVs containing DNA fragments, with a capacity to inhibit normal hematopoiesis. Thus, it is likely that leukemia cell-derived extranuclear DNA contributes to the suppression of normal hematopoiesis. This assumption prompted us to investigate the effects of extranuclear DNA on leukemic hematopoiesis, which shares many features with normal ones, and we demonstrated that extranuclear DNA accumulated in the cytoplasm of leukemia cells and suppressed leukemic hematopoiesis.

STING was originally identified as a central adaptor protein to sense cytoplasmic DNA. Several lines of evidence indicate that STING activation in HSCs can depress their hematopoietic capacity^16,17^. Consistently, we observed that a single exposure of leukemia cell-derived EVs to normal HSPCs reduced their in vitro colony formation in a STING-dependent manner. Moreover, we revealed that BMT caused less DNA damage in donor cells when STING-deficient HSPCs were used as donor cells in CML-bearing mice, compared with the use of WT HSPCs. Consequently, fatal anemia development was significantly delayed. Thus, it is plausible that leukemia cell-derived DNAs, which were transferred to the cytoplasm of normal hematopoietic cells, can disturb normal hematopoiesis in a STING pathway-dependent manner.

The potential cytotoxicity of extranuclear DNA^18^ prompted Takahashi et al. to elucidate the dynamics of extranuclear DNA, which were released from the nuclei of senescent fibroblasts^19^. They demonstrated that DNA accumulated in the cytoplasm of senescent fibroblasts was discarded after being incorporated into exosomes to maintain cellular homeostasis. Moreover, the repression of cytoplasmic DNases accelerated the senescence status and nuclear DNA damage, eventually inducing the accumulation of cytoplasmic DNA, which activated cytosolic DNA-sensor pathways^15^. Another study demonstrated that the prevention of autophagy-mediated degradation of cytoplasmic DNA activated DNA-sensor signaling, eventually enhancing DNA damage^14^. These observations suggest that autophagy can reduce the cytoplasmic DNA levels in addition to cytoplasmic DNases. Consistent with these findings, we revealed that inhibition of autophagosome formation efficiently induced cytoplasmic DNA accumulation, and subsequently, STING pathway-mediated cytotoxicity in leukemia cells. Given the moderate effects of an autolysosome formation inhibitor on cytotoxicity, autophagosomes may be mainly utilized to sequester cytoplasmic DNA, thereby concealing them from DNA sensing machinery to prevent cytotoxicity in leukemia cells.

We observed that STING pathway activation in leukemia cells resulted in the generation of ROS, which could be a main executor of the cytotoxicity induced by autophagosome inhibitors. STING pathway activation can stimulate IκB kinase and TANK-binding kinase 1 to activate two distinct transcription factors, NF-κB and IRF3, respectively, and both these factors can induce the expression of several antimicrobial and inflammation-related molecules, including ROS^20–22^. We observed that an autophagosome inhibitor enhanced the gene expression of *TNF-α*, *IL-12*, and *MCP-1*, in a STING pathway-dependent manner. In contrast, the gene expression of *ISG54* and *ISG56*, was augmented by an autophagosome inhibitor, even in the absence of STING pathway activation. Several lines of evidence indicate that NF-κB activation can regulate the gene expression of *TNF-α*, *IL-12*, and *MCP-1*^23^ and that the gene expression of *ISG54* and *ISG56* can be regulated by IRF-3^24^. Thus, autophagosome inhibitor-mediated STING pathway activation might induce ROS production mainly through the NF-κB pathway. However, as the loss of STING did not completely abrogate autophagy inhibitor-mediated cytotoxicity in leukemia cells, the contribution of STING-independent activation of IRF3 cannot be excluded.

We demonstrated that autophagosome inhibitors improved the survival of AML-bearing mice, suggesting the potential of these agents for AML treatment. Moreover, their therapeutic effects can be ascribed to their capacity to reduce AML cell proliferation by the action of ROS, which were generated through cytoplasmic DNA-mediated STING pathway activation. Cytoplasmic DNA accumulation is observed in various types of cancer cells^25,26^, and can be ascribed mainly to nuclear DNA release into the cytoplasm upon replication stress or DNA damage^27,28^. Thus, autophagosome formation inhibitor may be a potent therapeutic drug against various types of leukemias and other malignancies, which grow rapidly and are under replication stress. Moreover, as anti-cancer drugs and irradiation can cause DNA damage in cancer cells, these inhibitors may be able to supplement these therapies.

SBI-0206965 and MRT-68921 are inhibitors of a serine/threonine kinase, ULK-1, which can prevent autophagosome formation, and can inhibit several additional kinases^29^. Chen et al. reported that in addition to ULK1, MRT-68921 can inhibit NUAK family SNF1-like kinase 1 (NUAK1), an anti-oxidant molecule, and eventually can exert effective cytotoxicity in various solid cancer cell lines^30^. Moreover, Hwang et al. recently reported that both SBI-0206965 and MRT-68921 could induce degradation of internal tandem duplication mutations in the FLT3 tyrosine kinase receptor (FLT3-ITD) protein, thereby exerting cytotoxicity in FLT-ITD AML cells^31^. Thus, SBI-0206965- or MRT-68921-mediated inhibition of other kinases may also contribute to their cytotoxicity in leukemia cells. Nevertheless, a more precise study on the pharmacology of these agents is warranted to develop a novel strategy to treat leukemia.

## Supplementary information

Supplemental Material

## Data Availability

Raw and processed microarray data have been deposited in the GEO repository. The accession number is G1 (GEO reviewer link).
