# Supplementary material for: Cytoplasmic DNA accumulation preferentially triggers cell death of myeloid leukemia cells by interacting with intracellular DNA sensing pathway
Source: Cell Death Dis. 2021 Mar 26;12(4):322. doi: 10.1038/s41419-021-03587-x (PMC7997981; doi:10.1038/s41419-021-03587-x)
Supplement: Supplementary file 1 — Supplemental Material [file 41419_2021_3587_MOESM1_ESM.pdf]

Supplemental Figure Legends

Supplementary Figure 1. CML-bearing mice succumb to severe anemia after BMT therapy with a long latency. **(a)** Schema of BMT therapy for CML-bearing mice. **(b)** Chimerisms of GFP (BCR-ABL)-expressing residual leukemia cells and donor-derived cells in the biopsied LK^+ population after BMT therapy are shown. Data represent mean + SD from five (2w, 4w, 6w, and 8w after BMT therapy) or four (NT) independent experiments. NT shows the proportion of GFP^+ cells in untreated CML mice at 15 days after LIC transplantation. **(c)** Survival rates within 160 days after BMT therapy in CML mice. As a control, mice transplanted with normal BM cells instead of LICs were subjected to the BM transplantation. (control, n = 3; CML, n = 10) **(d)** Moribund mice were sacrificed and subjected to the detection of the residual leukemia cells. Expression of GFP and CD45.1 in the LK^+ cells harvested from BM and SP or that in the PB $CD11b^+$ myeloid cells are shown. Representative results from four independent experiments are shown here**.** **(e)** Hematological parameters (WBCs, RBCs, HGB, PLTs, and HCT) in PB within 8 weeks after BMT therapy and in the moribund mice (Mori). As a control, normal mice were subjected to the BM transplantation. Each symbol represents an individual mouse (n = 4).

Supplementary Figure 2. AML mouse-derived EVs inhibit colony formation of normal HSPCs. Colony formation of LKS^+ cells when they were co-cultured with or without EVs. The colony numbers are shown. Data represent mean ± SD from four independent experiments. *P < 0.01 by two-sided Student's t-test.

Supplementary Figure 3. CML SP-derived EVs contain *BCR-ABL* gene. *BCR-ABL* gene in the SP-derived EVs was detected using genomic PCR method. Normal SP-derived EVs were used as a negative control. Each lane represents an individual mouse-derived EVs.

Supplementary Figure 4. Intravesicular dsDNAs are detected in AML serum. Serum was harvested from mice 4 weeks after transplantation of AML cells along with normal BM cells. As a control, serum was harvested from mice transplanted with normal BM cells. EVs were purified using exoEasy Maxi Kit (QIAGEN). Protein concentration of EVs and intravesicular dsDNA concentration in AML serum were shown in **a** and **b**, respectively. Data represent mean ± SD from three independent experiments.

Supplementary Figure 5. Autophagy regulates the loading of dsDNAs into EVs. (a) dsDNA concentrations in the 7-day- or 10-day-cultured CML cell-derived EVs. Data represent mean ± SD from three independent experiments. *$P < 0.05$ by two-sided Student's *t*-test. **(b)** BCR-ABL$^+$ colony-forming cells were harvested at day 7 and 10. Nuclear and extranuclear dsDNAs were stained with Vybrant DyeCycle Green. Representative results from six independent experiments are shown**. (c)** BCR-ABL$^+$ colony-forming cells were harvested at day 7 of colony formation assay and were subsequently co-cultured with 1 or 5 μM rapamycin (LC Laboratories) or 5 or 10 μM SBI-0206965 (ULK1 inhibitor), in S-Clone medium supplemented with 1 % BSA, 100 ng/ml stem cell factor, 100 ng/ml thrombopoietin, 25 ng/ml *fms*-like tyrosine kinase-3 ligand, 10 ng/ml IL-6, and 10 ng/ml IL-3 for 2 days. Intravesicular dsDNA concentration in the culture supernatant was determined. DMSO was added in the culture medium as a vehicle control. Fold change between treatment and control is shown. Data represent mean ± SD from three or six independent experiments. **$P < 0.01$; *$P < 0.05$ by Tukey-Kramer test.

Supplementary Figure 6. Validation of autophagy inhibitors. Autophagosome was stained with DAPRed (DOJINDO) 16 h after treatment with 20 μM SBI-0206965 (SBI) or 20 μM hydroxychloroquine (HCQ). Representative results from eight photos are shown in the upper panels**.** Number of DAPRed$^+$ foci per each cell was analyzed with

Hybrid Cell Count software (Keyence). Data represent mean ± SD from eight photos. *$P < 0.05$ by Tukey-Kramer test.

Supplementary Figure 7. Cytotoxic assay of autophagy inhibitors. (a) Determination of cytotoxity of MRT-68921 against HL-60 cells ($n = 4$). **(b)** Determination of cytotoxity of hydroxychloroquine against leukemia cell lines ($n = 4$).

Supplementary Figure 8. shRNA KD efficiency. mRNA and protein expression of STING **(a)** and AIM2 **(b)** in each gene KD HL-60 cells.

Supplementary Figure 9. Attenuation of cytotoxicity in STING KD leukemia cells. (a) Determination of cytotoxity of MRT-68921 against shRNA control or STING KD HL-60 cells ($n = 4$). **(b)** Determination of cytotoxity of SBI-0206965 against shRNA control or STING KD THP-1 cells ($n = 4$). **$P < 0.01$ by two-sided Student's *t*-test.

Supplementary Figure 10. Attenuation of cytotoxicity of SBI-0206965 in cGAS KD AML cells. (a) mRNA and protein expression of cGAS in shRNA control or cGAS KD HL-60 cells. **(b)** Cytotoxic assay of SBI-0206965 against shRNA control or cGAS KD HL-60 cells ($n = 4$). *$P < 0.05$ by two-sided Student's *t*-test.

Supplementary Figure 11. Moderate effect of SBI-0206965 on the colony formation of normal mouse HSPCs. Colony formation of mouse LKS^+ cells when they were co-cultured with 5 μM SBI-0206965. As a control, cells were treated with DMSO. The colony numbers are shown. Data represent mean ± SD from three independent experiments. **$P < 0.01$ by two-sided Student's *t*-test.

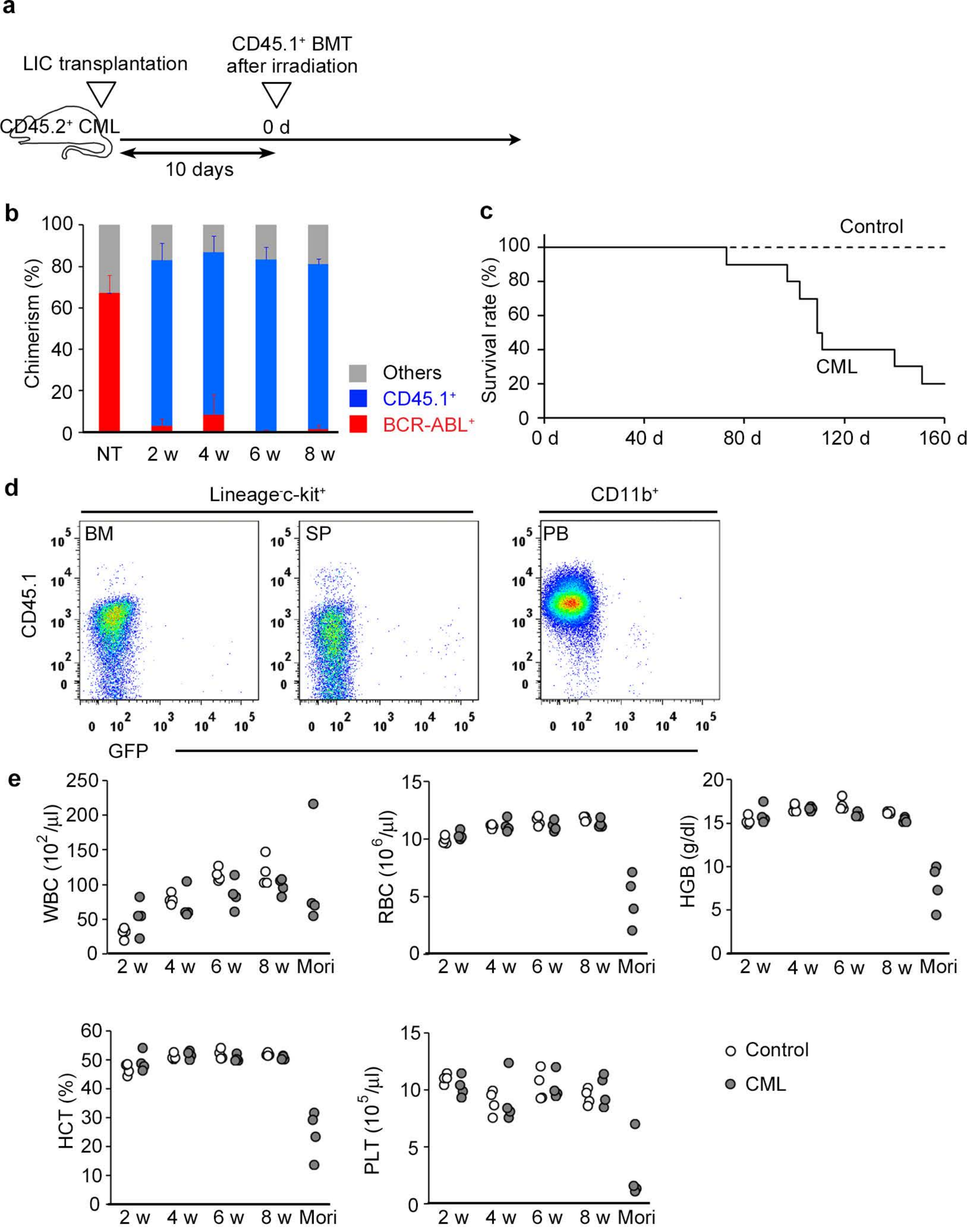

Supplementary Figure 1

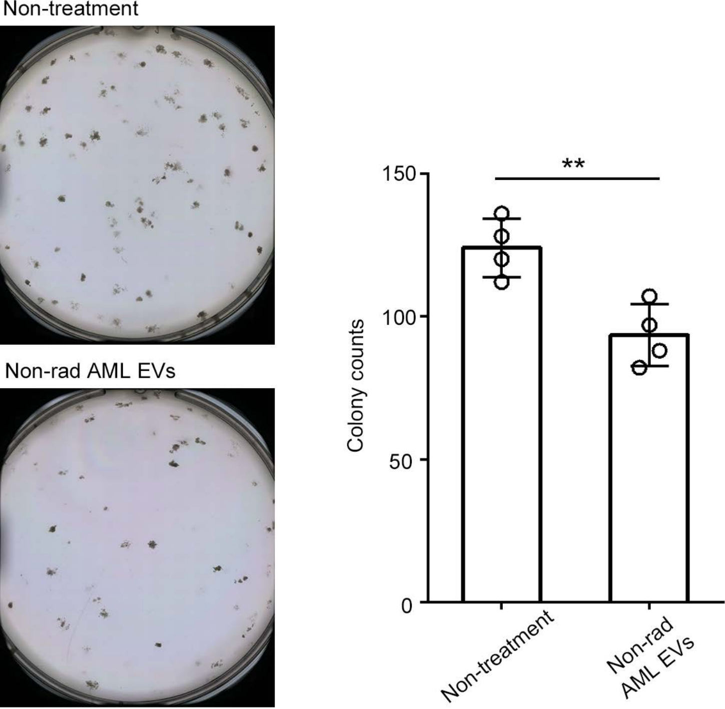

Supplementary Figure 2

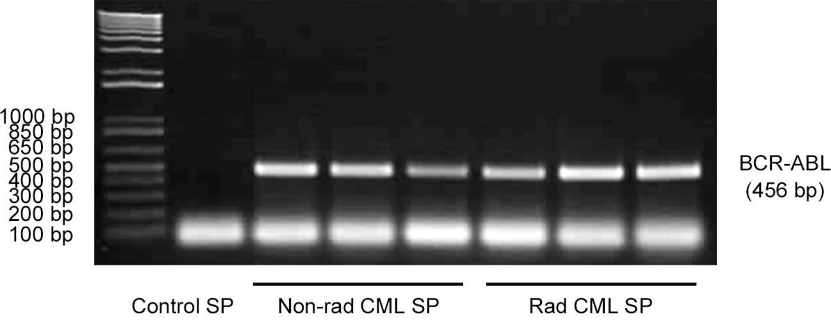

Supplementary Figure 3

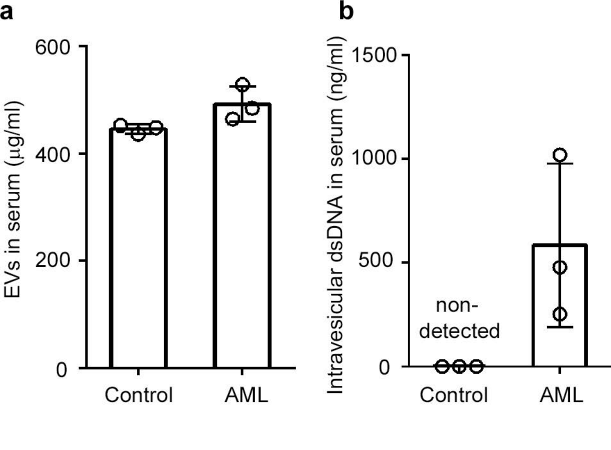

Supplementary Figure 4

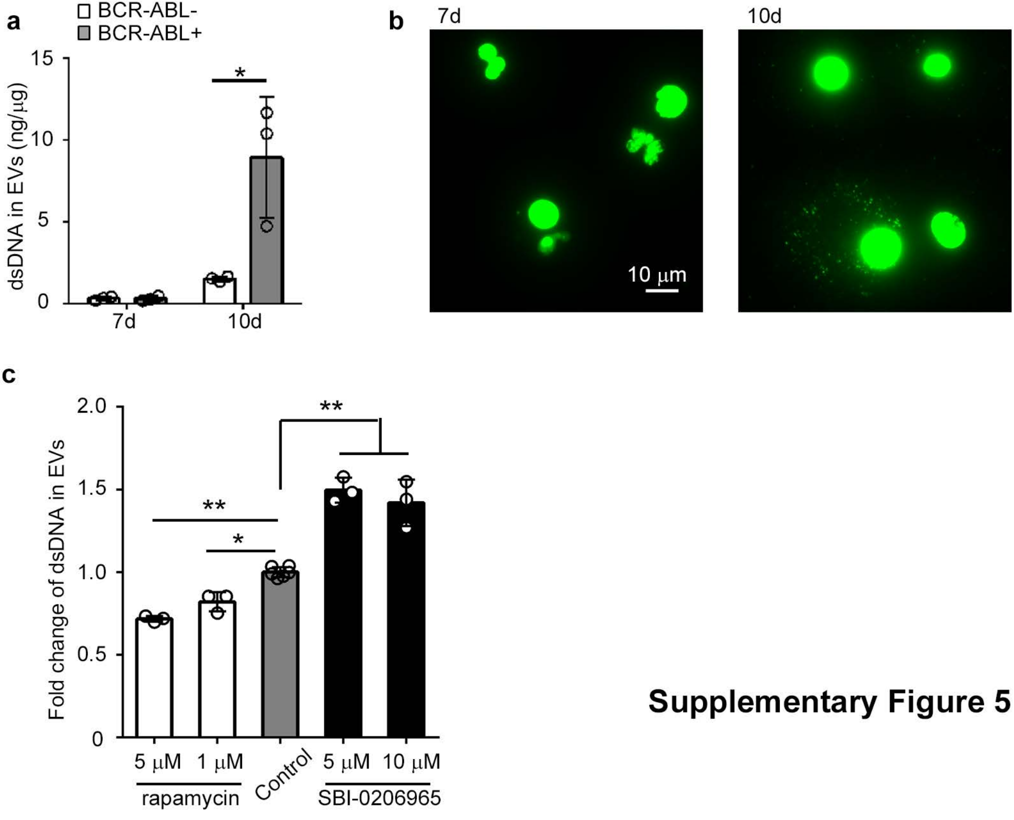

Supplementary Figure 5

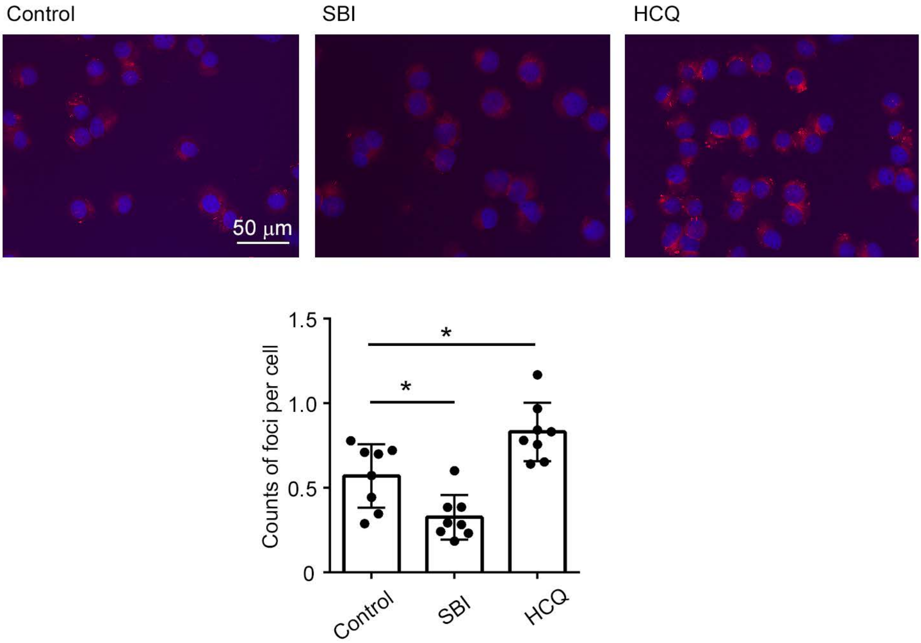

Supplementary Figure 6

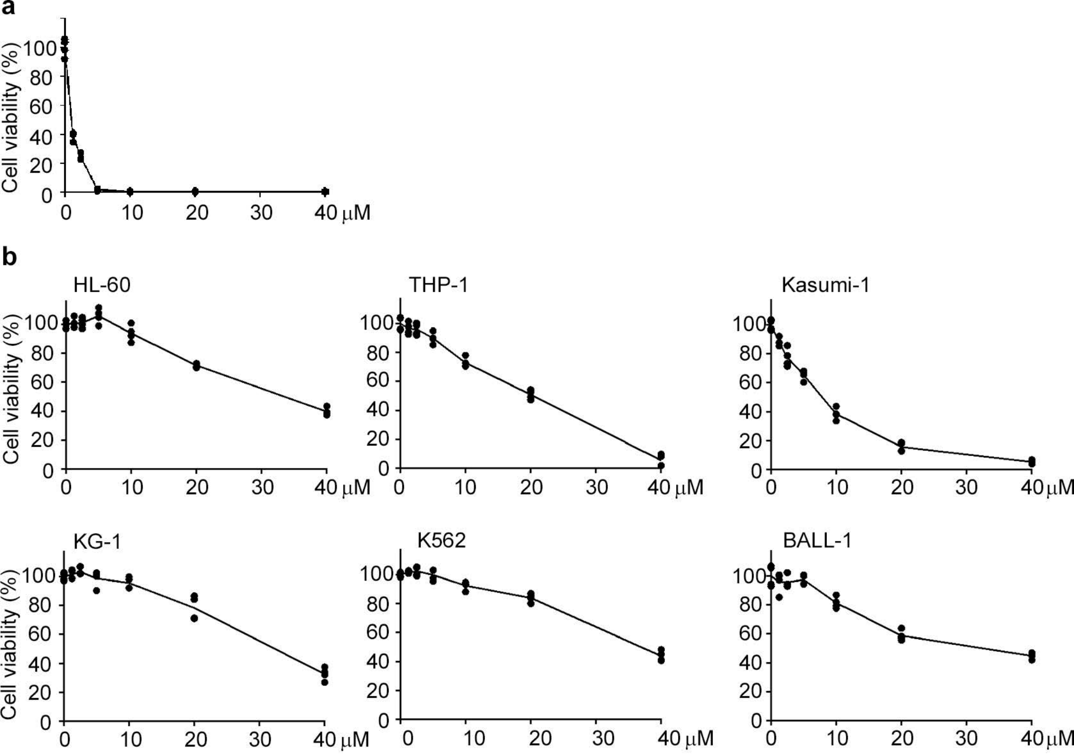

Supplementary Figure 7

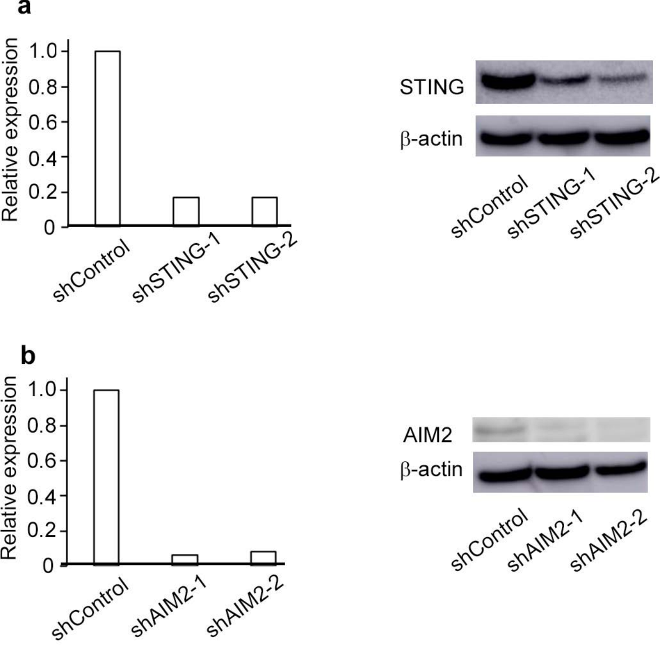

Supplementary Figure 8

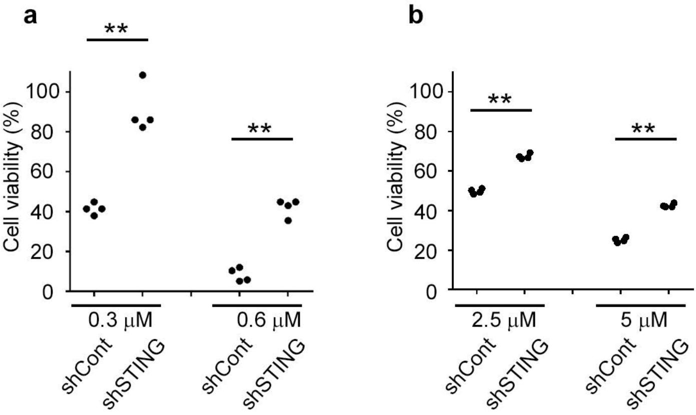

Supplementary Figure 9

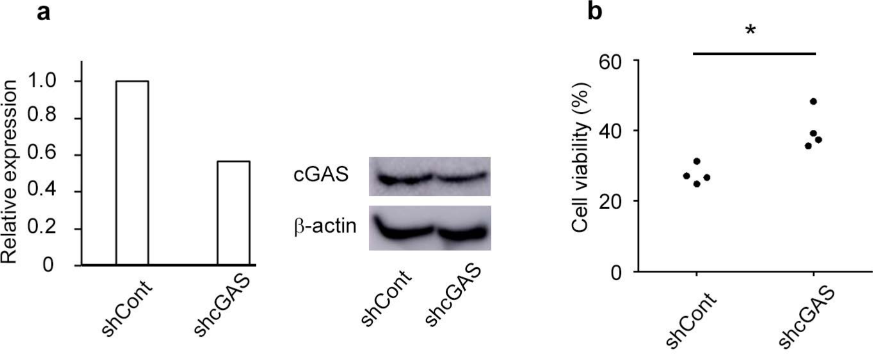

Supplementary Figure 10

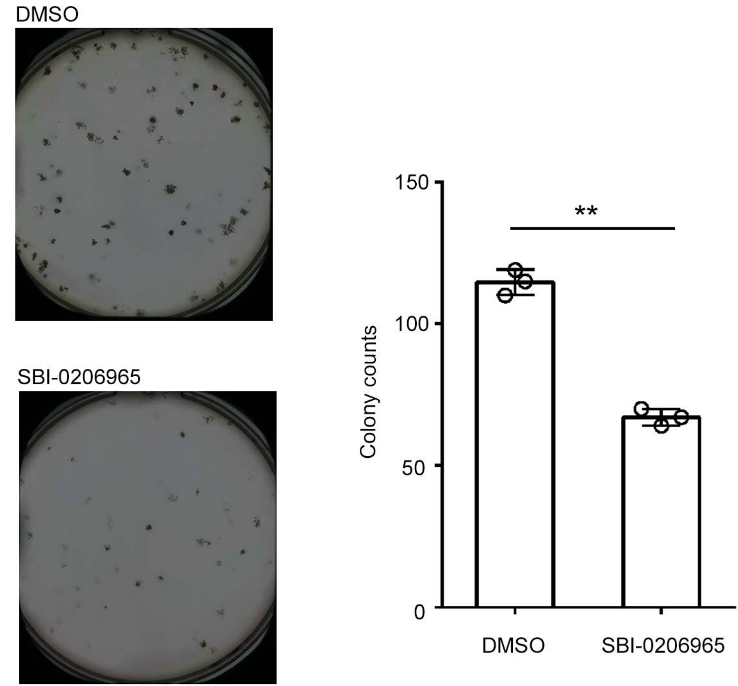

Supplementary Figure 11
